# MRA-based 3D-printed heart model—an effective tool in the pre-surgical planning of DORV

**DOI:** 10.1259/bjrcr.20150436

**Published:** 2016-07-28

**Authors:** Alpa Bharati, Swati Garekar, Vijay Agarwal, Suleman A Merchant, Narayan Solanki

**Affiliations:** ^1^Department of Radiology, LTMG Hospital, Mumbai, India; ^2^Department of Cardiac Imaging, NM Medical Center, Mumbai, India; ^3^Department of Pediatric Cardiology & Cardiac Surgery, Fortis Hospital, Mumbai, India

## Abstract

A three-dimensional (3D) printed heart model based on contrast-enhanced MR angiography data was obtained in an 8-month-old male child with double-outlet right ventricle. The model could successfully show the spatial relationship between the aortic annulus, the pulmonary valve and the ventricular septal defect. The patient underwent complete intracardiac repair based on the 3D model. MR angiography images could be successfully used to create a true-size 3D heart model, which significantly helped in assessing the routability of the ventricular septal defect to the aorta, leading to successful intracardiac repair in our patient.

## Background

Double-outlet right ventricle (DORV) is a complex congenital heart defect that requires detailed evaluation to assess the feasibility of complete intracardiac repair (ICR). Initial two-dimensional (2D) echocardiography establishes the diagnosis and status of the ventricles. In the presence of good biventricular size and function, and adequate sizes of the aorta and pulmonary arteries, biventricular ICR can offer better post-operative outcomes with fewer reinterventions than a palliative univentricular repair. However, in some cases, even though the above criteria are met, ICR can be difficult to perform if the intracardiac anatomy is complex and a potential baffle between the ventricular septal defect (VSD) and the aorta may cause obstruction in the right ventricle (RV) inflow or compromise the RV volume. Other factors that jeopardize ICR are significant straddling of the tricuspid valve, multiple chordae across the VSD and the distance of the VSD from the aortic and pulmonary valves.

In such patients, who are otherwise good subsets for ICR, the challenge is to assess the path of the potential baffle in terms of its length and the possibility of interference by the RV chordae during surgery and RV inflow obstruction or volume loss as a result of the baffle.

On 2D imaging modalities, such as echocardiography and MRI, an obvious obstruction to or by the potential baffle can be judged; however, in borderline cases, this becomes a major challenge. It is obviously impractical to surgically explore the child to check. A three-dimensional (3D) print model of the heart has tremendous potential to fill this loophole and provide excellent spatial information to allow pre-surgical planning and assess the possibility of ICR in these cases.

We present the case of an 8-month old male child with DORV who benefited from a basic 3D heart model printed using a cost-effective technique that helped in planning for ICR that was considered difficult based on the 2D echocardiography and MRI data.

## Case presentation

**Figure 1.**
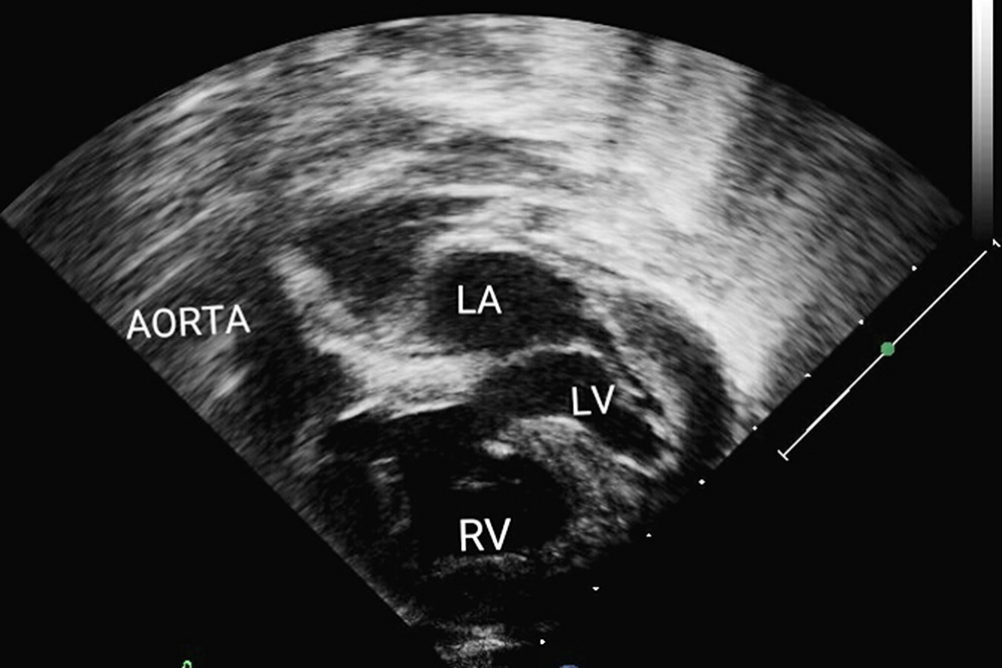
Transthoracic two-dimensional echocardiography showing the ventricular septal defect, aortic annulus and expected course of the baffle. LA, left atrium; LV, left ventricle; RV, right ventricle.

An 8-month-old infant weighing 6.8 kg presented with repeated chest infections and 94% oxygen saturation, measured by peripheral oximetry. Transthoracic echocardiography revealed a DORV with good biventricular size and function, mild pulmonary stenosis with a peak gradient of 28 mmHg and a large 0.8-cm-sized remote inlet VSD (Figure 1). The aortic valve was to the right and slightly anterior to the pulmonary valve. Surgical options of a staged palliation by pulmonary artery banding followed by Fontan *vs* a complete biventricular repair were considered. The latter appeared difficult owing to the long length of the baffle connecting the VSD to the aorta, possible interference of the anterior leaflet of the tricuspid valve along the baffle path and RV adequacy post baffle. For further clarity, it was decided to obtain a cardiac MRI and, if needed, a 3D model of the heart.

**Figure 2.**
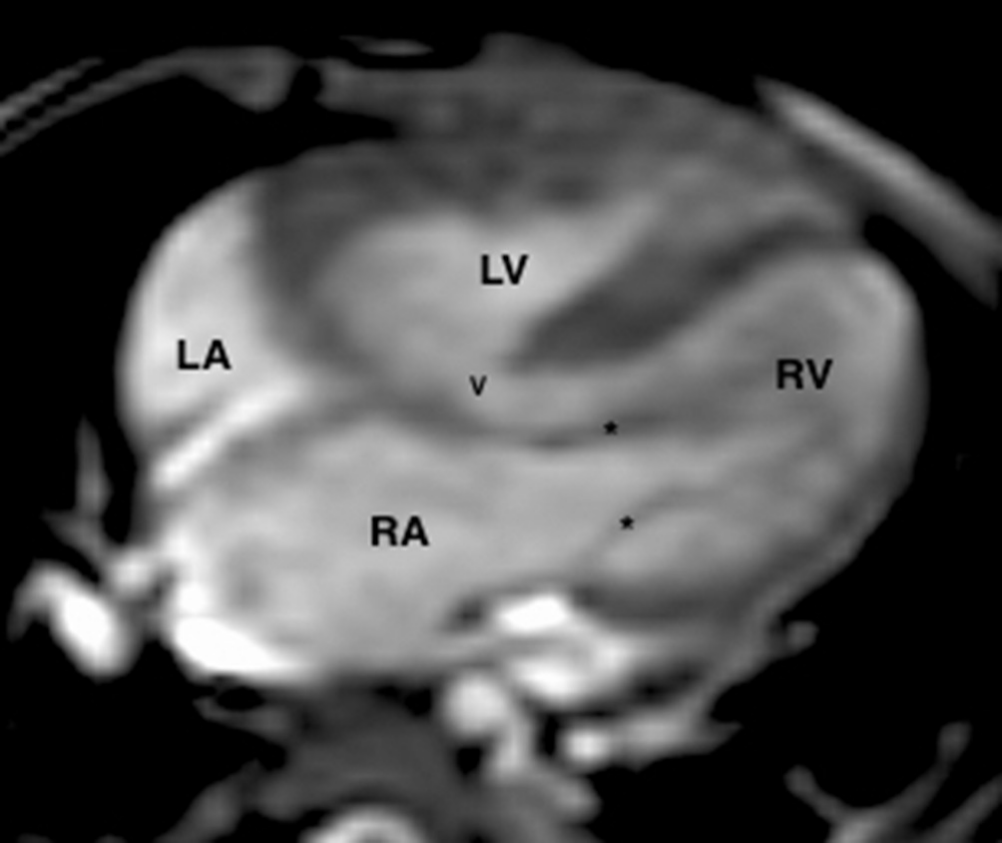
Cardiac MR image in the standard four-chamber view showing preserved RV volume (RV) and a large ventricular septal defect (v). The tricuspid leaflets (stars) are seen in proximity to the ventricular septal defect. LA, left atrium; LV, left ventricle; RA, right atrium.RV: right ventricle

The patient underwent cardiac MRI with contrast-enhanced MR angiography (MRA). The procedure was performed on a 3 T MRI scanner under short general anaesthesia using ketamine. Cine images were obtained onin the standard cardiac planes (Figure 2) followed by contrast-enhanced MRA using 2 ml of gadopentate dimeglumine (Magnevist, Zydus – German Remedies, India) injected *via* the left antecubital vein. Image acquisition was started once the contrast reached the RV. Images were acquired over 12 phases using free breathing sequence without electrocardiographic gating. The images were further analyzed on multiplanar reconstructions. The MRA images were used for 3D printing. Post-processing was performed using images in digital images and communication in medicine format (DICOM), which were then converted to stereolithography format for 3D printing. MR images were limited in resolution to allow clear demarcation of the ventricular chordae and the valve leaflets, which were therefore excluded in the final 3D model. Two models were printed, one made of plastic and another of sandstone. The cut planes in the model were planned along the short-axis plane in the former and the coronal plane, parallel to the interventricular septum, in the latter. The sandstone model and cuts parallel to the interventricular septum, which showed the en face view of the VSD, were best suited for further assessment compared with the short-axis plane. The 3D model could clearly depict the spatial orientation of the VSD to the aortic and pulmonary annuli (Supplementary Video A).^1^ The distance between these could be subjectively assessed and its effects on the RV inflow and volume could be very well estimated with the true-size model. Based on the model, it was concluded that the tricuspid leaflets would not interfere with the baffle and the possibility of complete ICR was considered. The patient underwent complete ICR after the model was inspected and was found to satisfy the criteria for RV volume, VSD size, distance of the VSD from the aortic annulus and the estimated length of the VSD–aorta baffle. On surgical exploration, all findings of the model were confirmed, except for the size of the VSD. The VSD appeared smaller in size cthan that on the model and needed to be enlarged before the baffle could be placed. There was no interference by the chordae or tricuspid leaflets and the baffle was successfully placed. The patient had an uneventful post-operative course and was discharged on day 10 post surgery.

## Discussion

3D-printed heart models for pre-surgical evaluation in complex congenital heart diseases have recently been performed using either CT scan or MRI data. 3D Models of the heart have been studied previously by Valverde et al^1^ and Liu et al.^2^ CT images have inherent high spatial resolution and can be used for performing a quick examination. However, it is constrained by radiation exposure, especially in infants and children, and the inability to provide functional data. On the other hand, cardiac MRI offers a radiation-free alternative but has lower spatial resolution. However, MRI has the advantage of providing functional information, especially of the RV in terms of right ventricular end diastolic volume, ejection fraction and stroke volume, which are important parameters in surgical decision making. When intracardiac structures have already been assessed well on 2D echocardiography and the main concern is the baffle path, its length and location, and relationship with the tricuspid annulus, MRA may be a preferred modality over CT imaging in DORV. Current literature demonstrates the use of both CT scan and MRI in obtaining 3D heart models of patients with complex congenital heart diseases.^1,2^ Liu et al^2^ prepared pre-operative 3D heart models in seven infants using low-dose cardiac CT scanning. They used prospective electrocardiographic gating with an acquisition window of 200 ms. Their study demonstrated high accuracy between the 3D models and intraoperative findings with the mean absolute error (3D *vs* real) in the size of septal defects being < 1 mm. Valverde et al^1^ on the other hand, studied 3D heart models of eight patients, of which four patients underwent CT scan and the remaining four underwent MRA. Preference of one modality over the other was not mentioned in their article. They compared the vascular diameter and size of the septal defects as seen on the CT scan and MRA images with those obtained on 3D models and found a variation of ±1.4 mm.^1^ Both the authors concluded that 3D printed models provide accurate representation of the real structure and significantly improve the understanding and the surgical approach^1,2^ and may potentially reduce the time required for surgery and complications.^2^

We chose MRI, as the primary goal of imaging was not only to obtain anatomical information but also acquire information on RV volume as there was concern of loss of RV volume post baffle. RV could be assessed accurately on MRI; in addition, it served to avoid radiation exposure. MRA data was sufficient to provide accurate spatial information about the relationship of the VSD to the pulmonary, aortic and tricuspid annuli. We feel that the imaging modality for 3D printing should be chosen with the aim of answering questions and doubts posed on 2D echo, which would heavily influence further surgical choices.

## Conclusions

MRA is a radiation-free, non-invasive imaging technique that can be used for creating a true-size 3D heart model. The MRA-data-based 3D sandstone heart model is cost-effective and can provide substantial intracardiac spatial information to accurately demonstrate the relationship of the VSD, aorta, pulmonary and tricuspid annulus for pre-surgical planning. It significantly reduces the communication gap between the radiologist, cardiologist and cardiac surgeon. In our case, based on the model, the patient could benefit from a complete ICR rather than a palliative univentricular repair.

We believe 3D printing can have a huge impact on pre-surgical planning and may result in many patients undergoing complete repair rather than palliation or *vice versa*. A larger case series is needed for further evaluation.

## Learning points

1. 3D printing in complex congenital heart disease, such as DORV, is an invaluable tool for pre-surgical assessment in establishing the feasibility of complete ICR.
2. 3D printing is particularly useful in those cases of DORV who fit the criteria for complete repair but are difficult candidates for ICR owing to a long or complex baffle route.

## Acknowledgment

We thank the technical support and efforts of Ms Firoza Kothari in developing the 3D model.

## Consent

Written informed consent was obtained from the patient for publication of this case report and any accompanying images.
